# The Impact of Intraoperative Position Changes on Hemodynamics and Cardiac Electrophysiological Balance Index in Patients with Severe Obesity Undergoing Laparoscopic Sleeve Gastrectomy

**DOI:** 10.1007/s11695-026-08497-5

**Published:** 2026-01-31

**Authors:** Fatma Celik, Recai Dagli, Ahmet Aksu, Murat Harman, Esef Bolat, İsmail Demirel, Gülsüm Altuntaş, Aysun Yıldız Altun

**Affiliations:** 1 Department of Anaesthesiology and Reanimation, Faculty of Medicine, Firat University, Elazig, Turkey; 2 Department of Anaesthesiology and Reanimation, Faculty of Medicine, Kırsehir Ahi Evran University, Kırsehir, Turkey; 3 Department of Cardiology, Faculty of Medicine, Fırat University, Firat University, Elazig, Turkey

**Keywords:** Laparoscopic sleeve gastrectomy, Severe obesity, Pneumoperitoneum, Patient positioning, Electrocardiography, Hemodynamics

## Abstract

**Background:**

Pneumoperitoneum and the reverse Trendelenburg (RT) position during laparoscopic sleeve gastrectomy (LSG) can induce autonomic instability and increase the risk of arrhythmias by reducing venous return. This study aimed to evaluate the impact of surgical positioning during LSG on autonomic cardiac function, using hemodynamic parameters and the cardiac electrophysiological balance index (iCEB = QT/QRS) as a biomarker.

**Methods:**

This prospective observational study included 66 patients with severe obesity who underwent LSG. Measurements were recorded at five distinct time points, corresponding to specific patient positioning during the procedure: P-baseline (before induction, supine), P1 (after induction, supine), P2 (after pneumoperitoneum, supine), P3 (during pneumoperitoneum, RT), and P4 (after desufflation, RT).

**Results:**

Systolic, diastolic, and mean arterial pressures (SAP, DAP, and MAP) significantly decreased at all positions compared to baseline (*p* < 0.001 for each). Compared to post-induction (P1), SAP values were substantially higher in the P2 and P4 positions (*p* < 0.05, *p* < 0.001, respectively). Heart rate significantly decreased at P1 compared to baseline (*p* < 0.004) and subsequently increased at P2 and P3 relative to P1 (*p* < 0.001 and *p* < 0.009, respectively). A notable increase in iCEB was observed at P4 when compared to P1, P2, and P3 (*p* < 0.003, *p* < 0.001, and *p* < 0.021, respectively). Despite these changes, iCEB values remained within the reference range across all measured positions.

**Conclusion:**

Despite the observed effects of positional changes and pneumoperitoneum on hemodynamic and cardiac electrical parameters during LSG, most patients tolerated these changes well. Crucially, iCEB values remained within the normal reference range throughout the procedure, indicating preserved cardiac autonomic regulation.

**Supplementary Information:**

The online version contains supplementary material available at 10.1007/s11695-026-08497-5.

## Key Points

• Positional changes during laparoscopic sleeve gastrectomy resulted in significant alterations in hemodynamic parameters and cardiac electrical activity in patients with severe obesity.

• After induction, the cardiac electrophysiological balance index (iCEB) initially demonstrated a significant decrease, then gradually increased, peaking statistically after desufflation in the reverse Trendelenburg position.

• Despite dynamic intraoperative changes, iCEB values consistently remained within the normal reference range (3.14–5.35) throughout the procedure, indicating preserved cardiac autonomic regulation.

**Supplementary Information:**

The online version contains supplementary material available at 10.1007/s11695-026-08497-5.

## Introduction

 Laparoscopic sleeve gastrectomy (LSG) is now the predominant bariatric procedure globally [1]. However, intraoperative hemodynamic stability and cardiac responsiveness are affected by a confluence of factors, including obesity-linked cardiovascular alterations, associated medical problems, pneumoperitoneum, elevated carbon dioxide levels, and the use of the reverse Trendelenburg (RT) position, all of which pose significant challenges for anesthetic management [2–5]. Pneumoperitoneum is a major cause of cardiovascular depression, as abdominal insufflation impairs systemic venous return, thereby decreasing preload and cardiac output while increasing afterload. Additionally, pressure from abdominal organs on the iliac veins may further limit venous return. Other factors affecting these physiological responses include hypercarbia, patient position, and intravascular volume status. While the RT position enhances alveolar ventilation, this position may also restrict venous return and lead to hemodynamic instability [3, 4, 6, 7]. Furthermore, the pneumoperitoneum and the RT position elevate sympathetic activity, increasing the risk of cardiac arrhythmias [8, 9].

Obesity has been identified as an independent determinant of increased cardiovascular morbidity and mortality [10–12]. Structural and functional cardiac alterations associated with obesity have been shown to promote electrical conduction abnormalities and repolarization disturbances, which may contribute to these adverse outcomes. Reports suggest that factors such as increased sympathetic activity, decreased baroreflex sensitivity, and autonomic imbalance in obesity may directly affect cardiac electrophysiological properties [2, 5, 11, 13, 14]. Therefore, the electrophysiological susceptibility in severe obesity patients may enhance arrhythmogenesis due to the combined effects of anesthesia, pneumoperitoneum, and positional changes during laparoscopic procedures. However, evidence regarding the implications of intraoperative position changes on electrocardiographic parameters during bariatric surgery is limited. To date, no study has evaluated the influence of intraoperative position changes during LSG on cardiac autonomic function in patients with severe obesity using the index of cardiac electrophysiological balance (iCEB).

The index of cardiac electrophysiologic balance, a non-invasive ECG-derived parameter defined as the QT/QRS ratio, serves as a surrogate marker of cardiac wavelength (λ = effective refractory period × conduction velocity) and is considered valuable for identifying susceptibility to malignant ventricular arrhythmias. The relationship between the effective refractory period and the QT interval further enhances the clinical value of iCEB [15, 16].

This study aims to evaluate the effects of changes in sequential position during LSG on cardiac autonomic function in patients with severe obesity through hemodynamic measurements and iCEB analysis.

## Materials and Methods

### Design of the Study and Ethical Considerations

This prospective observational study was approved by the Clinical Research Ethics Committee of Firat University (Approval No: 26146, dated 01/08/2024) and registered at ClinicalTrials.gov (Identifier: NCT06835166).

### Patient Inclusion and Exclusion Criteria

The study was conducted at Firat University Hospital between February 2025 and May 2025.

Inclusion criteria: (1) elective primary LSG surgery; (2) American Society of Anesthesiologists (ASA) physical health class III; (3) age ≥ 18 years; (4) body mass index (BMI) ≥ 40 kg/m^2^.

Exclusion criteria: (1) revision sleeve gastrectomy; (2) emergency LSG for complications (e.g., staple line leaks); (3) elective LSG accompanied by secondary surgery; (4) previous recurrent intra-abdominal surgeries; (5) patients with electrolyte imbalances; (6) multiple intubation attempts due to difficult intubation; (7) preoperative arrhythmia and ejection fraction < 30%; (8) thyroid gland diseases, renal failure, and hepatic failure; (9) patients requiring a tidal volume greater than 8 mL/kg.

### Practice

An experienced anesthesia team followed a standardized protocol. Patients were brought to the operating room without premedication. Patients underwent routine monitoring, including heart rate (HR), non-invasive blood pressure, peripheral oxygen saturation, body temperature, bispectral index (BIS; Dräger Vista 120 system, Covidien, USA), and pulse CO-oximetry (Masimo Rainbow SET^®^, Masimo Corporation, Irvine, CA, USA). They were also monitored with a 12-lead ECG (Cardioline^®^) to obtain electrocardiogram (ECG) measurements.

Preoxygenation was provided (100% O₂ at 4 L/min for 3 min). After induction with 2 mg/kg propofol, 1 µg/kg remifentanil, and 1 mg/kg rocuronium bromide, endotracheal intubation was performed. Drug doses were based on adjusted body weight (ABW) [ABW: ideal body weight (IBW) + 0.4 × (actual body weight - IBW)]. Mechanical ventilation was applied using a tidal volume of 8 mL/kg (IBW) and 8 cmH₂O positive end-expiratory pressure. Patients were ventilated with 4 L/min (50% O₂ − 50% air) and 2.5% sevoflurane for 10 min. Anesthesia was maintained using mid-flow techniques with 2 L/min (50% O₂/50% air) and 2.5–4.5% sevoflurane (target minimum alveolar concentration: 0.9–1.1). End-tidal carbon dioxide was maintained at 35 to 45 mmHg. Patients received remifentanil at 0.05–0.2 µg/kg/min (IBW), while BIS was maintained between 40 and 60.

Demographic characteristics such as age, gender, BMI, associated medical problems, medications, ASA physical status classification, duration of surgery and anesthesia, amount of intraoperative fluid given, and norepinephrine (NE) dose administered if needed were recorded. LSG was performed by the same surgical team with 15 mmHg CO₂ pneumoperitoneum in approximately 45° reverse Trendelenburg position.

Systolic (SAP), diastolic (DAP), and mean arterial pressure (MAP) measurements, along with HR and 12-lead ECG, were evaluated. Measurements were performed 5 min after the position change to ensure standardization, to allow the physiological response to settle after the position change, and to prevent exaggerated data.

Measurements were recorded at five distinct time points based on patient positioning during the procedure: P-baseline (before induction, supine), P1 (after induction, supine), P2 (pneumoperitoneum, supine), P3 (pneumoperitoneum, RT), and P4 (after desufflation, RT).

Sevoflurane was turned off in the last 10 min of the surgery. Before extubation, the gas flow rate was raised to 6 L/min. Ventilation was maintained manually with 80% O₂. Reversal of neuromuscular blockade was achieved with 2 mg/kg (ABW) sugammadex.

### Fluid and Vasopressor Management

At least two peripheral intravenous catheters (20G or larger) were placed before anesthesia induction, and a 500 mL bolus of Ringer’s lactate solution was administered. Maintenance fluid therapy was infused at 2 mL/kg/h of 0.9% NaCl solution, calculated based on actual body weight.

All hemodynamic interventions were guided by a predefined, goal-directed hemodynamic therapy (GHT) algorithm based on the MAP and Plethysmograph Variability Index (PVI). Mean arterial pressure was measured at five-minute intervals [17].

The hemodynamic protocol was as follows:

1. Stable Condition (PVI < 13% and MAP > 65 mmHg):

- A baseline infusion of 0.9% NaCl solution at 2 mL/kg/h was maintained.

2. Hypotension (Not Fluid Responsive) (PVI < 13% and MAP < 65 mmHg):

- Baseline fluid replacement (2 mL/kg/h) was continued.
- A 2 µg NE bolus was administered.
- If the same conditions persisted at the next assessment (5 min), the 2 µg NE bolus was repeated.
- If MAP remained below 65 mmHg in consecutive measurements, a continuous NE infusion was initiated at a rate of 0.1–0.4 µg/kg/min (NE concentration: 0.08 mg/mL).

3. Hypotension (Fluid Responsive) (PVI > 13% and MAP < 65 mmHg):

- A 250 mL crystalloid bolus was administered.
- If MAP persisted below 65 mmHg, an additional 2 µg NE bolus was given.
- If both PVI > 13% and MAP < 65 mmHg persisted in subsequent assessments, the 250 mL crystalloid bolus was repeated once.
- If MAP remained < 65 mmHg despite these interventions, a continuous NE infusion (0.1–0.4 µg/kg/min, 0.08 mg/mL) was initiated, and fluid therapy was continued until PVI decreased be low 13%.

### Electrocardiographic Evaluation

Demographic and clinical information for each patient was recorded on the ECG device before the operation. The device was set to 50 mm/sec and 1 mV. A cardiologist, blinded to the study’s procedural positions, evaluated all ECGs. First, iCEB (QT/QRS), the study’s primary outcome, was assessed. Additionally, the T peak-to-peak (Tpe) interval, QT, heart rate-corrected QT (QTc), QRS, Tpe/QTc, heart rate-corrected iCEB (iCEBc = QTc/QRS), and Tpe/QT parameters were analyzed. QT interval and QRS duration were assessed in leads II and V5, and the longest measurements were noted. The QTc interval was calculated using the Bazett formula based on heart rate: QTc = QT/√RR.

### Statistical Analysis

Power analysis was performed using the G*Power 3.1.9.2 statistical package program; *n* = 66, effect size (f) = 0.25, α = 0.05, and power (1-β) = 0.95. The data were analyzed using IBM SPSS Statistics, version 22.0.0 (IBM Corp., Armonk, NY, USA). Demographic and intraoperative variables were presented as mean ± standard deviation or frequency counts, as appropriate. Hemodynamic changes and ECG measurements were expressed as mean ± standard deviation. Data normality was assessed using the Kolmogorov-Smirnov and Shapiro-Wilk tests.

The variables QT, QTc, Tpe/QTc, iCEB (QT/QRS), iCEBc (QTc/QRS), and Tpe/QT were analyzed using a one-way repeated measures ANOVA as they followed a normal distribution. The Bonferroni test was subsequently applied for post hoc analysis. The variables Tpe and QRS were found to be non-normally distributed. For their overall comparison across positions, the non-parametric Friedman test was employed. If the Friedman test indicated a significant difference (*P* < 0.05), the Wilcoxon signed-rank test with Bonferroni correction was planned for post hoc pairwise comparisons. A P-value of < 0.05 was considered statistically significant.

## Results

**Fig. 1:**
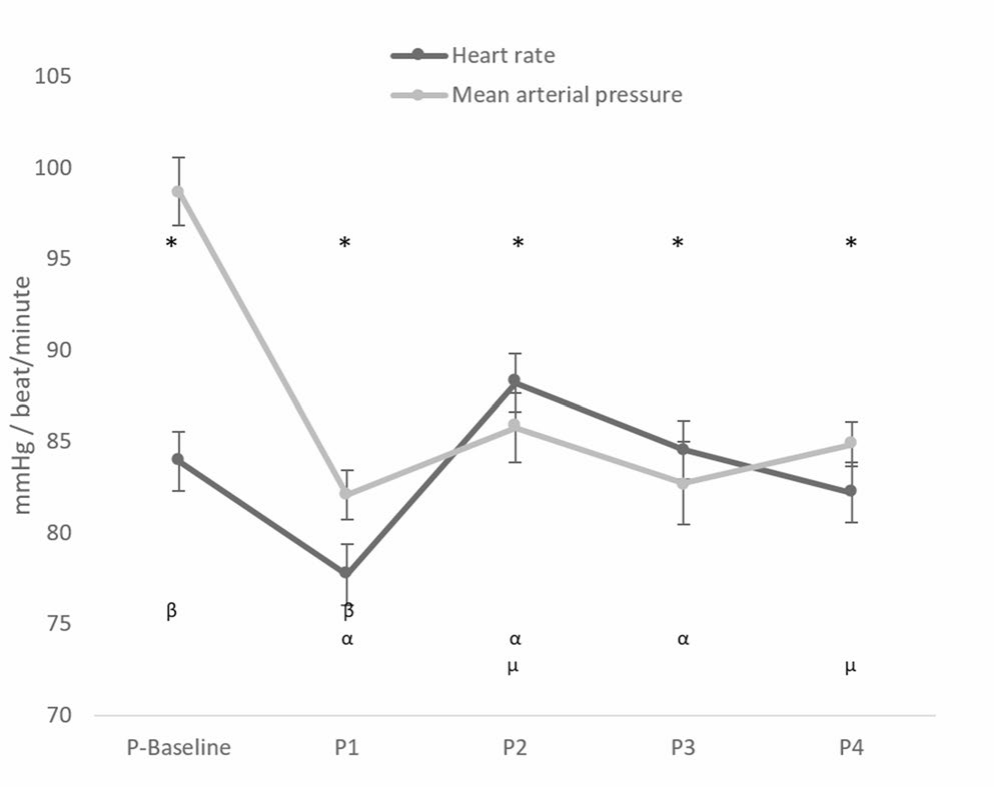
Intraoperative hemodynamics. Data are presented as mean ± standard deviation. P-Baseline: Before anesthesia induction + supine position; P1: After induction of anesthesia + supine position; P2: Pneumoperitoneum + supine position; P3: Pneumoperitoneum + reverse Trendelenburg position; P4: Pneumoperitoneum desufflation + reverse Trendelenburg position. Statistical comparisons were performed using one-way repeated measures ANOVA followed by Bonferroni post-hoc test. P-value < 0.05 was considered statistically significant Statistical Significance Markers: MAP: *: Significantly different from P-Baseline (for P1, P2, P3, P4) HR: β: Significantly different from P-Baseline (for P1) α: Significantly different from P1 (for P2, P3) µ: Significantly different from P2 (for P4)

The primary outcome was the change in iCEB across different surgical positions. A previously published study reported an iCEB value of 4.24 (reference range: 3.14–5.35) [15]. In our research, iCEB analysis was based on this reference range. The study initially recruited 85 patients. After excluding 10 patients for not meeting the inclusion criteria and nine due to baseline iCEB values falling outside the reference range, 66 patients remained for the final analysis. Table 1 summarizes the demographic characteristics and intraoperative data, and Table 2 presents the hemodynamic data (SAP, DAP, MAP, and HR) across the five measurement points. Systolic arterial pressure was statistically lower in all positions compared to the baseline value (all *p* < 0.001). Compared to P1, SAP was significantly higher in P2 and P4 (*p* < 0.05 and *p* < 0.001, respectively). Conversely, DAP and MAP values showed significant reductions from baseline in all positions (all *p* < 0.001). The variations in HR and MAP observed in different positions are displayed in Fig. 1. HR was lower than baseline in position P1 (*p* < 0.004). In contrast, HR significantly increased in positions P2 and P3 relative to P1 (*p* < 0.001, *p* < 0.009, respectively). Additionally, HR in position P4 was lower than in position P2 (*p* < 0.04).

**Table 1:** Patient demographics and intraoperative variables

Characteristics	*N* = 66
Age (years)	35.77 ± 10.80
Gender (female/male)	39/27
BMI (kg/m^2^)	45.38 ± 5.78
ASA PS	III
Operation time (min)	84.65 ± 18.85
Anesthesia time (min)	103.03 ± 19.71
Fluid (mL)	1411.36 ± 308.45
Noradrenaline (push/infusion)	12/7
Hypertension	10
Diabetes mellitus	5
Pulmonary diseases	4
Antihypertensive drugs	10
Antidiabetic drugs	5
Bronchodilator drugs	1

*BMI* Body mass index; *ASA PS* American Society of Anesthesiologists physical status.

classification; The data are presented as mean ± standard deviation.

**Table 2:** Arterial pressures and heart rate changes

	*P*-Baseline	P1	P2	P3	P4
SAP	136.56 ± 2.36^a^	109.53 ± 1.64^a, b^	117.79 ± 2.64^a, b^	112.83 ± 2.93^a^	118.83 ± 1.64^a, b^
DAP	74.09 ± 1.63^a^	65.20 ± 1.25^a^	66.38 ± 1.72^a^	64.50 ± 1.92^a^	63.73 ± 1.18^a^
MAP	98.70 ± 1.86^a^	82.08 ± 1.35^a^	85.76 ± 1.92^a^	82.73 ± 2.26^a^	84.86 ± 1.23^a^
HR	83.92 ± 1.64^a^	77.70 ± 1.68^a, b^	88.20 ± 1.59^b, d^	84.55 ± 1.58^b^	82.21 ± 1.65^d^

Values are presented as mean ± standard deviation. *SAP* Systolic arterial pressure; *DAP* Diastolic arterial pressure; *MAP* Mean arterial pressure; *HR* Heart rate; *P-Baseline* Before anesthesia induction + supine position; P1: After induction of anesthesia + supine position; P2: Pneumoperitoneum + supine position; P3: Pneumoperitoneum + reverse Trendelenburg position; P4: Pneumoperitoneum desufflation + reverse Trendelenburg position. Statistical comparisons were performed using one-way repeated measures ANOVA followed by Bonferroni post-hoc test. P-value < 0.05 was considered statistically significant.

SAP: a: Significantly different from P-Baseline (for P1, P2, P3, P4). b: Significantly different from P1 (for P2, P4).

DAP: a: Significantly different from P-Baseline (for P1, P2, P3, P4).

MAP: a: Significantly different from P-Baseline (for P1, P2, P3, P4).

HR: a: Significantly different from P-Baseline (for P1). b: Significantly different from P1 (for P2, P3). d: Significantly different from P2 (for P4).

**Fig. 2:**
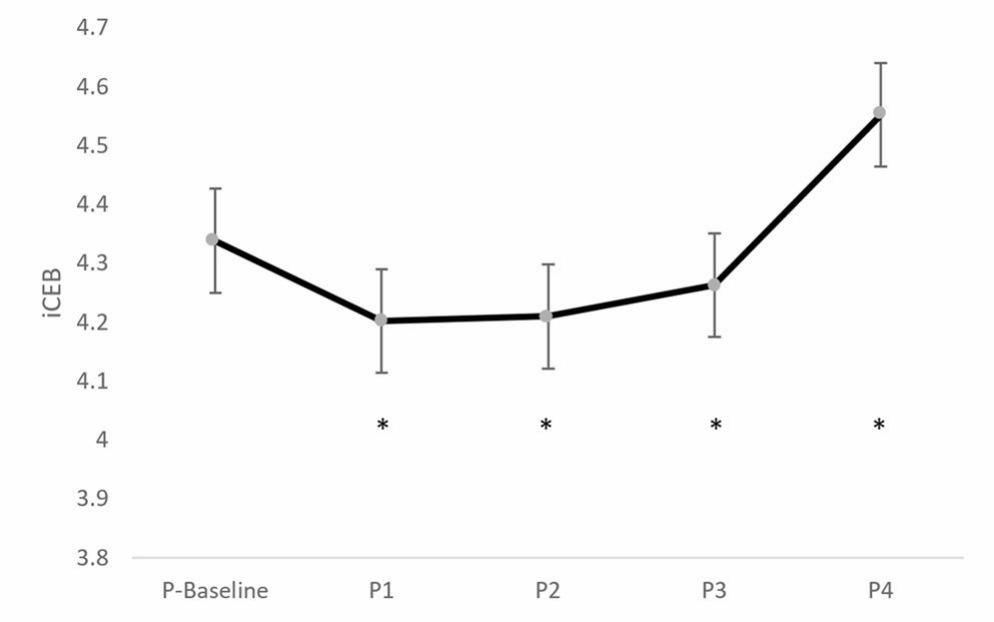
Change in average iCEB values ​​according to positions. Data are presented as mean ± standard deviation. iCEB: Index of cardiac electrophysiological balance; P-Baseline: Before anesthesia induction + supine position; P1: After induction of anesthesia + supine position; P2: Pneumoperitoneum + supine position; P3: Pneumoperitoneum + reverse Trendelenburg position; P4: Pneumoperitoneum desufflation + reverse Trendelenburg position. Statistical comparisons were performed using one-way repeated measures ANOVA followed by Bonferroni post-hoc test. P-value < 0.05 was considered statistically significant Statistical Significance Markers: iCEB: *: Significantly different from P4 (for P1, P2, P3)

The changes in mean iCEB values across positions are shown in Fig. 2. Accordingly, compared to P1, P2, and P3, position P4 displayed significantly elevated iCEB values (*p* < 0.003, *p* < 0.001, *p* < 0.021, respectively). QT duration reached its highest value at P4 compared to the other positions (all *p* < 0.001). Moreover, QT values were significantly higher in position P3 relative to P1 (*p* < 0.002), with P1 measurements notably lower than baseline (*p* < 0.006). QTc duration was also considerably higher in position P4 than in the other positions (*p* < 0.001, *p* < 0.009, *p* < 0.002, *p* < 0.026, respectively) (Table 3). Baseline, P2, and P3 positions showed significantly higher Tpe/QTc ratios compared to P4 (*p* < 0.01, *p* < 0.006, *p* < 0.016, respectively) (Table 3). A notable elevation in the iCEBc ratio was recorded at position P4 relative to baseline (*p* < 0.013). Table 3 indicates that the Tpe/QT ratio was significantly higher in all other positions compared to position P4 (*p* < 0.002, *p* < 0.001, *p* < 0.001, *p* < 0.001, *p* < 0.008, respectively).

**Table 3:** Electrocardiographic parameters across positions

	*P*-Baseline	P1	P2	P3	P4	*p*
Tpe	74.47 ± 2.44	71.82 ± 1.98	74.46 ± 2.39	74.47 ± 2.00	69.55 ± 2.34	0.179^*^
QT	370.15 ± 4.29^$^©	353.33 ± 3.46^$&^©	363.26 ± 4.62©	370.30 ± 4.64^&^©	388.79 ± 4.92©	< 0.001^**^
QTc	434.94 ± 4.50©	443.42 ± 3.91©	440.09 ± 4.87©	445.09 ± 4.27©	459.62 ± 4.28©	< 0.001^**^
QRS	86.80 ± 1.61	85.97 ± 1.70	88.24 ± 1.77	88.70 ± 1.72	87.18 ± 1.71	0.283^*^
Tpe/QTc	0.17 ± 0.01©	0.16 ± 0.01	0.17 ± 0.01©	0.17 ± 0.01©	0.15 ± 0.01©	0.004^**^
iCEB (QT/QRS)	4.34 ± 0.08	4.20 ± 0.08©	4.21 ± 0.09©	4.26 ± 0.09©	4.55 ± 0.01©	0.001^**^
iCEBc (QTc/QRS)	5.10 ± 0.09©	5.28 ± 0.11	5.18 ± 0.12	5.14 ± 0.11	5.40 ± 0.12©	0.018^**^
Tpe/QT	0.20 ± 0.01©	0.20 ± 0.01©	0.21 ± 0.01©	0.20 ± 0.01©	0.18 ± 0.01©	< 0.001^**^

^*^ Friedman Test ^**^ ANOVA Test

Values are presented as mean ± standard deviation. A P-value of < 0.05 was considered statistically significant

Tp-e: T wave peak-to-end; QTc: heart rate-corrected QT interval; iCEB: index of cardiac electrophysiological balance; iCEBc: corrected iCEB; P-Baseline: Before anesthesia induction + supine position; P1: After induction of anesthesia + supine position; P2: Pneumoperitoneum + supine position; P3: Pneumoperitoneum + reverse Trendelenburg position; P4: Pneumoperitoneum desufflation + reverse Trendelenburg position

QT: ©: Significantly different from P4 (for P-Baseline, P1, P2, P3). &: Significantly different from P3 (for P1). $: Significantly different from P1 (for P-Baseline).

QTc: ©: Significantly different from P4 (for P-Baseline, P1, P2, P3).

Tpe/QTc: ©: Significantly different from P4 (for P-Baseline, P2, P3).

iCEB (QT/QRS): ©: Significantly different from P4 (for P1, P2, P3).

iCEBc (QTc/QRS): ©: Significantly different from P4 (for P-Baseline).

Tpe/QT: ©: Significantly different from P4 (for P-Baseline, P1, P2, P3).

A total of 12 patients received at least one 2 µg NE push, and 7 patients required an NE infusion. Intraoperative details of norepinephrine administration, fluid boluses, cardiac rhythm status, and associated surgical positions—all applied according to the GHT algorithm—are presented in Table 4.

**Table 4:** Intraoperative norepinephrine administration, fluid boluses, cardiac rhythm status, and associated patient positions according to the goal-directed hemodynamic therapy algorithm

Patient	NE Infusion (µg/kg/min)	Number of NE Pushes (2 µg each)	Total NE Push (µg)	Push Position	Fluid Bolus (250 mL)	Bradyarrhythmia
1	0.1–0.3	2	4	P2	–	No
2	0.1–0.3	2	4	P2/P3	–	No
3	–	1	2	P2	–	No
4	–	2	4	P2	–	No
5	–	1	2	P3	P2 (1 bolus)	No
6	–	1	2	P2	P2 (1 bolus)	No
7	–	1	2	P2	–	No
8	0.1–0.3	2	4	P2	–	No
9	0.1–0.3	2	4	P3	–	No
10	0.1–0.4	2	4	P2/P3	–	Yes
11	0.1–0.3	1	2	P2	P2 (two boluses)	No
12	0.1–0.3	1	2	P3	P3 (two boluses)	No

*NE* Norepinephrine; *PVI* Plethysmograph Variability Index; *MAP* Mean Arterial Pressure; P2: Pneumoperitoneum + supine position; P3: Pneumoperitoneum + reverse Trendelenburg position.

## Discussion

This study demonstrates that surgical positioning and pneumoperitoneum during LSG significantly alter hemodynamic and cardiac electrical parameters. A significant hemodynamic suppression was observed following anesthesia induction, evidenced by reductions in SAP, DAP, and MAP relative to baseline. These changes likely result from the cardio-depressant effects of anesthetic agents and the physiological alterations caused by increased intra-abdominal pressure and positional adjustments. The P1 position showed the most pronounced decreases in HR, SAP, DAP, and MAP, consistent with the immediate effects of induction. Although these parameters partially increased in the P2 and P4 positions compared to P1, SAP levels remained below baseline, possibly due to reduced venous return and altered arterial tone. Structural and functional cardiac abnormalities commonly observed in individuals with obesity are linked to systemic and pulmonary hypertension, resulting from increased intravascular volume, elevated cardiac output, and enhanced ventricular strain. These cardiovascular changes, combined with limited cardiac reserve, elevate the risk of hypotension during anesthesia induction and increase susceptibility to perioperative fluid overload [2, 6, 10, 13, 18]. In contrast, the observed increases in heart rate during the supine and RT positions with pneumoperitoneum likely reflect autonomic imbalance and sympathetic activation. Conversely, the heart rate reduction in the P4 position may indicate reactivation of vagal tone and decreased sympathetic drive due to the reduction in intra-abdominal pressure. These findings highlight the need for individualized intraoperative monitoring, as the hemodynamic response to pneumoperitoneum and positional changes may vary significantly between patients. Hemodynamic parameters typically return to baseline rapidly after desufflation in healthy individuals; however, this process may take up to 65 min in those with cardiovascular disease. These findings suggest that increased intra-abdominal pressure and patient positions create more hemodynamic stress in these individuals, indicating they may be more sensitive to surgical interventions [4].

Previous studies have reported varying hemodynamic responses to laparoscopic surgery in patients with severe obesity. Aloni et al. suggested that younger age and a lower incidence of systemic diseases may contribute to hemodynamic stability in these patients [19]. In our study, hemodynamic changes remained relatively stable, possibly due to similar patient characteristics; however, some patients required low-dose noradrenaline infusion. Gaszyński et al. observed adverse hemodynamic compromise associated with pneumoperitoneum compared to open surgery, while Nguyen et al. reported good tolerance of pneumoperitoneum in patients with severe obesity [7, 20]. Our study demonstrates that the hemodynamic effects of patient positions are well tolerated and emphasizes the need for pharmacological treatment when necessary.

We present the first evaluation of the influence of different surgical positions on cardiac function during LSG in patients with severe obesity using iCEB. Previous studies have examined how pneumoperitoneum and RT positioning during laparoscopy influence cardiac repolarization, focusing on parameters such as the QT and QTc intervals in patients with non-severe obesity [8, 21]. The iCEB is considered an indicator of the interplay between cardiac depolarization and repolarization and has been utilized as a marker to assess the risk of drug-related ventricular arrhythmias [15, 16]. Therefore, the iCEB was preferred because it enables a more comprehensive assessment of ventricular electrical balance. While we utilized the well-established iCEB reference range [15], it should be noted that specific normal values for patients with severe obesity are currently not defined, underscoring the need for further validation studies in this cohort.

The findings indicate that anesthesia induction, positional changes, and pneumoperitoneum significantly affect cardiac electrical activity in patients with severe obesity. Following induction (P1), a marked decrease in iCEB was observed, followed by a gradual increase in subsequent positions, ultimately peaking at position P4 in a statistically significant manner. Despite these variations, iCEB values remained within the general reference range (3.14–5.35) throughout the procedure. Simultaneously, QTc duration also exhibited notable alterations. While the baseline QTc duration was approximately 435 ms, values exceeded the commonly accepted threshold of 440 ms in all subsequent positions, reaching approximately 460 ms at P4. The high iCEB and QTc values recorded in the P4 position suggest that these changes may be attributed to the RT position and the final period of surgery. This implies the cumulative effect of an average anesthesia duration of 103 min on cardiac repolarization. Additionally, this state may be connected to increased repolarization dispersion and prolonged intraventricular conduction time, which could indicate a heightened risk of ventricular arrhythmias. Notably, QTc prolongation is widely recognized as a marker of arrhythmic risk. However, iCEB provides a more comprehensive assessment, as it reflects both the depolarization and repolarization phases of the cardiac cycle. Therefore, monitoring iCEB may offer a more sensitive indicator of subclinical electrical instability, particularly in high-risk populations, such as severe obesity patients undergoing laparoscopic surgery. Our study indicates a significant increase in QTc and iCEBc values at P4 (relative to P-baseline). The accumulation of hemodynamic stress up to the P4 position, combined with the effects of the RT position, may have contributed to this change. This finding implies that cardiac electrical activity does not return to baseline five minutes after the cessation of pneumoperitoneum.

Several studies have reported that a pneumoperitoneum pressure of 15 mmHg is a threshold beyond which cardiac output begins to decrease [4, 7, 22]. Indeed, increased vagal tone due to pneumoperitoneum has been shown to cause bradyarrhythmia and even asystole. Even among young, healthy individuals with intra-abdominal pressure < 12 mmHg, the incidence of bradyarrhythmia has been found to range from 14% to 27% [4]. In our study, although noradrenaline infusion was administered at a low dose to seven patients, clinical bradyarrhythmia was noted in only one patient. In this patient, iCEB values were: P-baseline: 4.75; P1: 4.75; P2: 5.00; P3: 5.50; and P4: 4.40. The notably prolonged durations of surgery (165 min) and anesthesia (185 min) may have contributed to the occurrence of arrhythmia. Additionally, regardless of the anesthesia technique, both anesthetic agents and adjuvants may influence cardiac repolarization, either directly or indirectly, by altering ECG parameters. This suggests that the study population overall tolerated pneumoperitoneum and positional changes [23].

### Clinical Implications and Future Perspectives

#### Clinical Significance for Patient Safety

Our study identifies two primary clinical implications for intraoperative patient safety in during LSG. First, iCEB has the potential to serve as a comprehensive, integrative safety signal. The most critical finding is the coexistence of marked QTc prolongation (up to 460 ms at P4), which traditionally signals increased arrhythmic risk, and iCEB remaining within its normal reference range (3.14–5.35) throughout the surgical procedure. This observation suggests that, despite the significant electrical changes, the combined stress of pneumoperitoneum and positioning did not compromise the overall ventricular electrical stability, indicating an adequate compensatory mechanism. Therefore, from a patient safety perspective, iCEB has the potential to serve as a valuable, integrated monitor compared with QTc alone and could provide critical reassurance about the low risk of acute malignant ventricular arrhythmia.

Second, our findings reaffirm the critical role of hemodynamic stabilization in preserving cardiac electrophysiological balance. The GHT applied in this process played a significant role. This approach, by rapidly managing hypotension with NE push/infusion or fluid replacement based on fluid responsiveness, ensured optimal hemodynamic stability throughout the critical P2/P3 phases. This circumstance prevented excessive fluid loading and kept cardiovascular stress tolerable. This management contributed to the preservation of both the iCEB values and stable hemodynamic metrics observed.

#### Benefits for Anesthesiologists in the Field

Our results offer two key practical takeaways for anesthesiologists managing in LSG. Primary among these is the need for increased electrophysiological vigilance. Practitioners should note that the (RT) position post-desufflation (P4) was associated with the peak cardiac electrical change (indicated by the highest QTc and iCEB values), despite the relief of intra-abdominal pressure. This observation may challenge the conventional assumption that cardiovascular stress immediately concludes upon desufflation. This circumstance renders continuous and vigilant monitoring of ECG parameters, particularly QTc, prudent, and our data suggest that iCEB could serve as a valuable, complementary, and integrated metric for comprehensive risk assessment.

Equally important is preparedness for hemodynamic management. Our positional analysis clearly identified P2 and P3 as critical moments for hemodynamic intervention. This finding suggests the necessity for having vasopressors (e.g., 2 µg NE push doses) and fluid boluses readily available for potential hypotension immediately prior to the introduction of pneumoperitoneum and patient positioning. This preparedness enables the rapid correction of hypotension, preserves cerebral and myocardial perfusion, and helps maintain cardiac electrical balance.

The potential of iCEB requires overcoming technological challenges for successful translation into routine clinical practice. Data necessary for iCEB calculation and continuous, high-fidelity QTc monitoring are not yet fully integrated into standard anesthesia monitoring systems. Consequently, the clinical adoption of iCEB as a routine metric will depend on the development of next-generation, integrated monitoring devices.

#### Suggested Future Trials to Deepen the Impact

1. Index of Cardiac Electrophysiological Balance Threshold Validation: A large, prospective randomized trial is recommended to establish the optimal iCEB threshold for predicting major adverse postoperative cardiac events in severely obese patients undergoing non-cardiac surgery. Such a study would transition iCEB from an observational research tool to a clinically actionable predictive index that could guide perioperative risk assessment.
2. Hemodynamic Support Timing (P2/P3 Phases): Future research could focus on exploring the effect of early hemodynamic support strategies on iCEB during the P2 and P3 phases. These studies may help to determine whether intervention thresholds guided by iCEB monitoring create a clinically significant difference in maintaining cardiac stability compared to current standard hemodynamic protocols.
3. Interventions to Attenuate P4 Position QTc and iCEB Peaks: Further prospective studies are suggested to test the efficacy of targeted interventions specifically at the P4 position (where peak QTc and iCEB values were observed). These trials could explore whether specific treatments (e.g., modification of CO_2_ insufflation pressure or the use of a vasoactive agent) can mitigate this electrical peak, thereby potentially improving cardiac stability during emergence from anesthesia.

#### Limitations

There are some limitations to our research. First, the patients had a mean BMI of 45.38 ± 5.78 and were generally stable, without severe cardiorespiratory-associated medical problems. These findings may therefore apply only to this specific population. Second, the sample size was relatively small, which may affect the generalization of the results. However, the power analysis (1-β = 0.95) demonstrated sufficient statistical power. Third, a constraint on the interpretation of absolute iCEB values is that the reference range utilized in this study [15] has not been specifically validated for the unique physiological profile of the severe obesity population. Fourth, ECG measurements were obtained only at the 5th minute after each position application, without continuous monitoring throughout each position. Finally, the variables used in the study, such as anesthesia techniques, fluid management, and pharmacological treatment, were standard practices and were not evaluated as covariates. Therefore, this investigation could not determine the extent to which these factors contributed to the observed effects on hemodynamic parameters and ECG results.

## Conclusions

Patient positioning and pneumoperitoneum during LSG for severe obesity significantly alter hemodynamic parameters and cardiac electrical activity. Nevertheless, iCEB values remained within normal limits, indicating preserved cardiac autonomic function and adequate adaptation in most patients. Specifically, iCEB, by integrating both depolarization and repolarization phases, offered a more comprehensive assessment of ventricular electrical balance than traditional single-parameter ECG measurements, proving valuable in this high-risk population. These findings underscore the critical need for vigilant hemodynamic and cardiac electrical monitoring during LSG in patients with severe obesity, guiding anesthesiologists in optimizing perioperative management and warranting further investigation into long-term cardiac outcomes.

## Supplementary Information

Below is the link to the electronic supplementary material.

Supplementary Material 1 (PPTX 360 KB) 

Supplementary Material 2 (DOCX 20.8 KB)

## Data Availability

No datasets were generated or analysed during the current study.
