# Supplementary material for: The Impact of Intraoperative Position Changes on Hemodynamics and Cardiac Electrophysiological Balance Index in Patients with Severe Obesity Undergoing Laparoscopic Sleeve Gastrectomy
Source: Obes Surg. 2026 Jan 31;36(3):1210–9. doi: 10.1007/s11695-026-08497-5 (PMC13038688; doi:10.1007/s11695-026-08497-5)
Supplement: Supplementary file 1 — Supplementary Material 1 (PPTX 360 KB) [file 11695_2026_8497_MOESM1_ESM.pptx]

## Slide 1
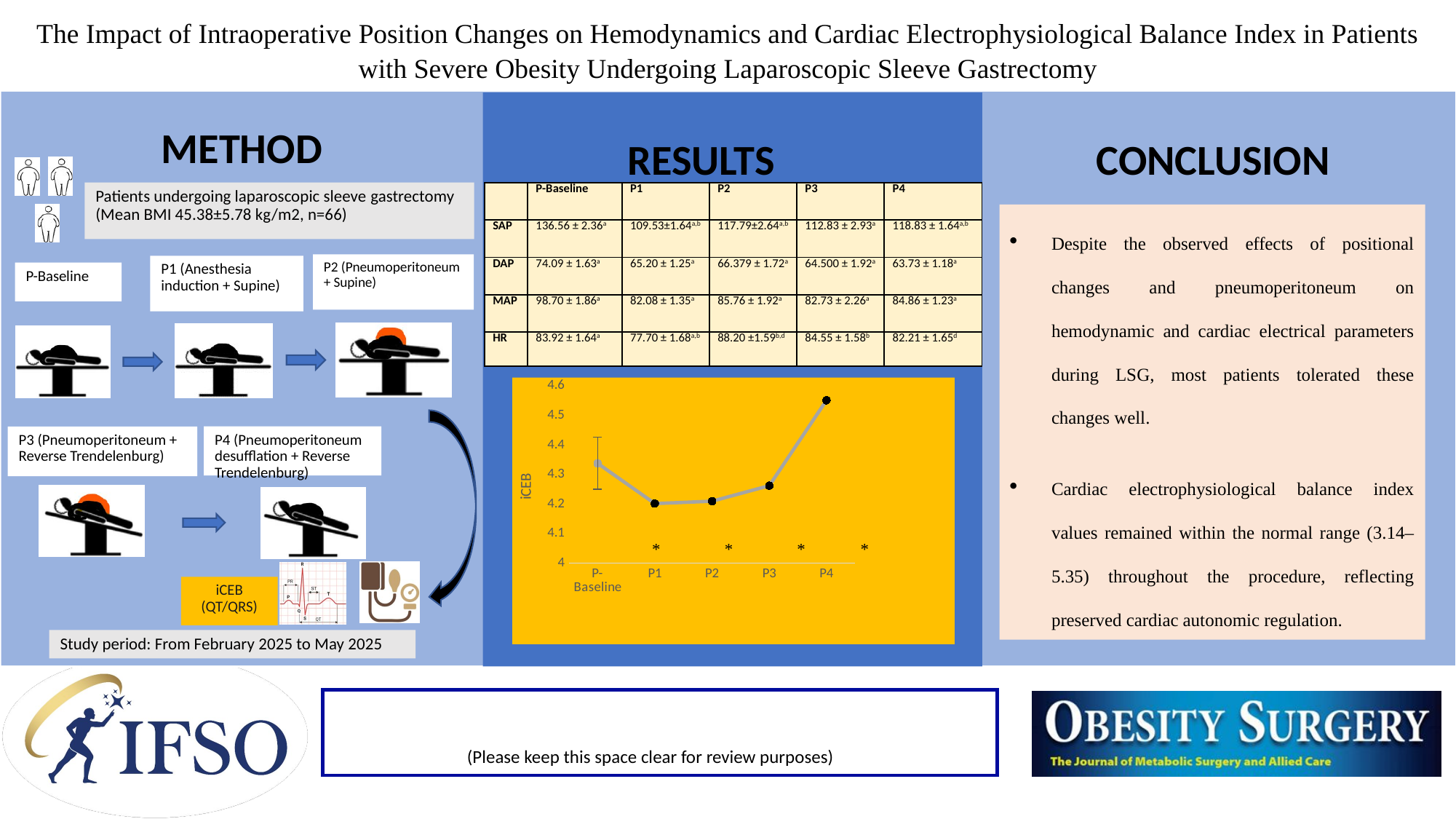

The Impact of Intraoperative Position Changes on Hemodynamics and Cardiac Electrophysiological Balance Index in Patients with Severe Obesity Undergoing Laparoscopic Sleeve Gastrectomy
P1 (Anesthesia induction+Supine)
 METHOD
RESULTS
CONCLUSION
Patients undergoing laparoscopic sleeve gastrectomy (Mean BMI 45.38±5.78 kg/m2, n=66)
| | P-Baseline | P1 | P2 | P3 | P4 |
| --- | --- | --- | --- | --- | --- |
| SAP | 136.56 ± 2.36a | 109.53±1.64a,b | 117.79±2.64a,b | 112.83 ± 2.93a | 118.83 ± 1.64a,b |
| DAP | 74.09 ± 1.63a | 65.20 ± 1.25a | 66.379 ± 1.72a | 64.500 ± 1.92a | 63.73 ± 1.18a |
| MAP | 98.70 ± 1.86a | 82.08 ± 1.35a | 85.76 ± 1.92a | 82.73 ± 2.26a | 84.86 ± 1.23a |
| HR | 83.92 ± 1.64a | 77.70 ± 1.68a,b | 88.20 ±1.59b,d | 84.55 ± 1.58b | 82.21 ± 1.65d |
Despite the observed effects of positional changes and pneumoperitoneum on hemodynamic and cardiac electrical parameters during LSG, most patients tolerated these changes well.
Cardiac electrophysiological balance index values remained within the normal range (3.14–5.35) throughout the procedure, reflecting preserved cardiac autonomic regulation.
P2 (Pneumoperitoneum + Supine)
P1 (Anesthesia induction + Supine)
P-Baseline
### Chart
| Category | |
|---|---|
| P-Baseline | 4.338084449409091 |
| P1 | 4.2016717620151525 |
| P2 | 4.209711121348486 |
| P3 | 4.262393652424242 |
| P4 | 4.551662042606062 |
P4 (Pneumoperitoneum desufflation + Reverse Trendelenburg)
P3 (Pneumoperitoneum + Reverse Trendelenburg)
iCEB (QT/QRS)
Study period: From February 2025 to May 2025
(Please keep this space clear for review purposes)
