# Supplementary material for: The Impact of Intraoperative Position Changes on Hemodynamics and Cardiac Electrophysiological Balance Index in Patients with Severe Obesity Undergoing Laparoscopic Sleeve Gastrectomy
Source: Obes Surg. 2026 Jan 31;36(3):1210–9. doi: 10.1007/s11695-026-08497-5 (PMC13038688; doi:10.1007/s11695-026-08497-5)
Supplement: Supplementary file 2 — Supplementary Material 2 (DOCX 20.8 KB) [file 11695_2026_8497_MOESM2_ESM.docx]

Table 3. Electrocardiographic parameters across positions

|  | **P-Baseline** | **P1** | **P2** | **P3** | **P4** | **p** |
| --- | --- | --- | --- | --- | --- | --- |
| **Tpe** | 74.47 ± 2.44 | 71.82 ± 1.98 | 74.46 ± 2.39 | 74.47 ± 2.00 | 69.55 ± 2.34 | 0.179^*^ |
| **QT** | 370.15 ± 4.29^$©^ | 353.33 ± 3.46^$&©^ | 363.26 ± 4.62^©^ | 370.30 ±4.64^&©^ | 388.79 ± 4.92^©^ | <0.001^**^ |
| **QTc** | 434.94 ± 4.50^©^ | 443.42 ± 3.91^©^ | 440.09 ± 4.87^©^ | 445.09 ± 4.27^©^ | 459.62 ± 4.28^©^ | <0.001^**^ |
| **QRS** | 86.80 ± 1.61 | 85.97 ± 1.70 | 88.24 ± 1.77 | 88.70 ± 1.72 | 87.18 ± 1.71 | 0.283^*^ |
| **Tpe/QTc** | 0.17 ± 0.01^©^ | 0.16 ± 0.01 | 0.17 ± 0.01^©^ | 0.17 ± 0.01^©^ | 0.15 ± 0.01^©^ | 0.004^**^ |
| **iCEB (QT/QRS)** | 4.34 ± 0.08 | 4.20 ± 0.08^©^ | 4.21 ± 0.09^©^ | 4.26 ± 0.09^©^ | 4.55 ± 0.01^©^ | 0.001^**^ |
| **iCEBc (QTc/QRS)** | 5.10 ± 0.09^©^ | 5.28 ± 0.11 | 5.18 ± 0.12 | 5.14 ± 0.11 | 5.40 ± 0.12^©^ | 0.018^**^ |
| **Tpe/QT** | 0.20 ± 0.01^©^ | 0.20 ± 0.01^©^ | 0.21 ± 0.01^©^ | 0.20 ± 0.01^©^ | 0.18 ± 0.01^©^ | <0.001^**^ |

**^*^ Friedman Test ^**^ ANOVA Test**

Values are presented as mean ± standard deviation. A P-value of <0.05 was considered statistically significant.

**Tp-e:** T wave peak-to-end; **QTc:** heart rate-corrected QT interval; **iCEB:** index of cardiac electrophysiological balance; **iCEBc:** corrected iCEB; **P-Baseline:** Before anesthesia induction + supine position; **P1:** After induction of anesthesia + supine position; **P2:** Pneumoperitoneum + supine position; **P3:** Pneumoperitoneum + reverse Trendelenburg position; **P4:** Pneumoperitoneum desufflation + reverse Trendelenburg position.

**QT:**

**©**: Significantly different from P4 (for P-Baseline, P1, P2, P3).

**&**: Significantly different from P3 (for P1).

**$**: Significantly different from P1 (for P-Baseline).

**QTc:**

**©**: Significantly different from P4 (for P-Baseline, P1, P2, P3).

**Tpe/QTc:**

**©**: Significantly different from P4 (for P-Baseline, P2, P3).

**iCEB (QT/QRS):**

**©**: Significantly different from P4 (for P1, P2, P3).

**iCEBc (QTc/QRS):**

**©**: Significantly different from P4 (for P-Baseline).

**Tpe/QT:**

**©**: Significantly different from P4 (for P-Baseline, P1, P2, P3).

Table 3.1. P-values for pairwise comparison of the (Tpe) parameter across patient positions

| **Tpe** | **Measurement values** | **P-Baseline**  **(p-value)** | **P1**  **(p-value)** | **P2**  **(p-value)** | **P3**  **(p-value)** | **P4**  **(p-value)** |
| --- | --- | --- | --- | --- | --- | --- |
| **P-Baseline** | 74.47 ± 2.44 | -- | 1.000 | 1.000 | 1.000 | 0.581 |
| **P1** | 71.82 ± 1.98 | 1.000 | -- | 1.000 | 1.000 | 1.000 |
| **P2** | 74.46 ± 2.39 | 1.000 | 1.000 | -- | 1.000 | 0.291 |
| **P3** | 74.47 ± 2.00 | 1.000 | 1.000 | 1.000 | -- | 0.331 |
| **P4** | 69.55 ± 2.34 | 0.581 | 1.000 | 0.290 | 0.330 | -- |

Table 3.2. P-values for pairwise comparison of the (QT) parameter across patient positions

| **QT** | **Measurement values** | **P-Baseline**  **(p-value)** | **P1**  **(p-value)** | **P2**  **(p-value)** | **P3**  **(p-value)** | **P4**  **(p-value)** |
| --- | --- | --- | --- | --- | --- | --- |
| **P-Baseline** | 370.15 ± 4.29 | -- | 0.006 | 1.000 | 1.000 | 0.001 |
| **P1** | 353.33 ± 3.46 | 0.006 | -- | 0.131 | 0.002 | < 0.001 |
| **P2** | 363.26 ± 4.62 | 1.000 | 0.131 | -- | 0.601 | < 0.001 |
| **P3** | 370.30 ± 4.64 | 1.000 | 0.002 | 0.601 | -- | < 0.001 |
| **P4** | 388.79 ± 4.92 | 0.001 | < 0.001 | < 0.001 | < 0.001 | -- |

Table 3.3. P-values for pairwise comparison of the (QTc) parameter across patient positions

| **QTc** | **Measurement values** | **P-Baseline**  **(p-value)** | **P1**  **(p-value)** | **P2**  **(p-value)** | **P3**  **(p-value)** | **P4**  **(p-value)** |
| --- | --- | --- | --- | --- | --- | --- |
| **P-Baseline** | 434.94 ± 4.50 | -- | 1.000 | 1.000 | 0.278 | < 0.001 |
| **P1** | 443.42 ± 3.91 | 1.000 | -- | 1.000 | 1.000 | 0.009 |
| **P2** | 440.09 ± 4.87 | 1.000 | 1.000 | -- | 1.000 | 0.002 |
| **P3** | 445.09 ± 4.27 | 0.278 | 1.000 | 1.000 | -- | 0.026 |
| **P4** | 459.62 ± 4.28 | < 0.001 | 0.009 | 0.002 | 0.026 | -- |

Table 3.4. P-values for pairwise comparison of the (QRS) parameter across patient positions

| **QRS** | **Measurement values** | **P-Baseline**  **(p-value)** | **P1**  **(p-value)** | **P2**  **(p-value)** | **P3**  **(p-value)** | **P4**  **(p-value)** |
| --- | --- | --- | --- | --- | --- | --- |
| **P-Baseline** | 86.80 ± 1.61 | -- | 1.000 | 1.000 | 1.000 | 1.000 |
| **P1** | 85.97 ± 1.70 | 1.000 | -- | 0.871 | 0.319 | 1.000 |
| **P2** | 88.24 ± 1.77 | 1.000 | 0.871 | -- | 1.000 | 1.000 |
| **P3** | 88.70 ± 1.72 | 1.000 | 0.319 | 1.000 | -- | 1.000 |
| **P4** | 87.18 ± 1.71 | 1.000 | 1.000 | 1.000 | 1.000 | -- |

Table 3.5. P-values for pairwise comparison of the (Tpe/QTc) parameter across patient positions

| **Tpe/QTc** | **Measurement values** | **P-Baseline**  **(p-value)** | **P1**  **(p-value)** | **P2**  **(p-value)** | **P3**  **(p-value)** | **P4**  **(p-value)** |
| --- | --- | --- | --- | --- | --- | --- |
| **P-Baseline** | 0.17 ± 0.01 | -- | 0.473 | 1.000 | 1.000 | 0.010 |
| **P1** | 0.16 ± 0.01 | 0.473 | -- | 1.000 | 1.000 | 0.177 |
| **P2** | 0.17 ± 0.01 | 1.000 | 1.000 | -- | 1.000 | 0.006 |
| **P3** | 0.17 ± 0.01 | 1.000 | 1.000 | 1.000 | -- | 0.016 |
| **P4** | 0.15 ± 0.01 | 0.010 | 0.177 | 0.006 | 0.016 | -- |

Table 3.6. P-values for pairwise comparison of the (iCEB: QT/QRS) parameter across patient positions

| **iCEB (QT/QRS)** | **Measurement values** | **P-Baseline**  **(p-value)** | **P1**  **(p-value)** | **P2**  **(p-value)** | **P3**  **(p-value)** | **P4**  **(p-value)** |
| --- | --- | --- | --- | --- | --- | --- |
| **P-Baseline** | 4.34 ± 0.08 | -- | 0.770 | 1.000 | 1.000 | 0.098 |
| **P1** | 4.20 ± 0.08 | 0.770 | -- | 1.000 | 1.000 | < 0.001 |
| **P2** | 4.21 ± 0.09 | 1.000 | 1.000 | -- | 1.000 | 0.003 |
| **P3** | 4.26 ± 0.09 | 1.000 | 1.000 | 1.000 | -- | 0.021 |
| **P4** | 4.55 ± 0.01 | 0.098 | < 0.001 | 0.003 | 0.021 | -- |

Table 3.7. P-values for pairwise comparison of the (iCEBc (QTc/QRS)) parameter across patient positions

| **iCEBc (QTc/QRS)** | **Measurement values** | **P-Baseline**  **(p-value)** | **P1**  **(p-value)** | **P2**  **(p-value)** | **P3**  **(p-value)** | **P4**  **(p-value)** |
| --- | --- | --- | --- | --- | --- | --- |
| **P-Baseline** | 5.10 ± 0.09 | -- | 0.648 | 1.000 | 1.000 | 0.034 |
| **P1** | 5.28 ± 0.11 | 0.648 | -- | 0.819 | 1.000 | 1.000 |
| **P2** | 5.18 ± 0.12 | 1.000 | 0.819 | -- | 1.000 | 0.081 |
| **P3** | 5.14 ± 0.11 | 1.000 | 1.000 | 1.000 | -- | 0.171 |
| **P4** | 5.40 ± 0.12 | 0.034 | 1.000 | 0.081 | 0.171 | -- |

Table 3.8. P-values for pairwise comparison of the (Tpe/QT) parameter across patient positions

| **Tpe/QT** | **Measurement values** | **P-Baseline**  **(p-value)** | **P1**  **(p-value)** | **P2**  **(p-value)** | **P3**  **(p-value)** | **P4**  **(p-value)** |
| --- | --- | --- | --- | --- | --- | --- |
| **P-Baseline** | 0.20 ± 0.01 | -- | 1.000 | 1.000 | 1.000 | 0.008 |
| **P1** | 0.20 ± 0.01 | 1.000 | -- | 1.000 | 1.000 | < 0.001 |
| **P2** | 0.21 ± 0.01 | 1.000 | 1.000 | -- | 1.000 | < 0.001 |
| **P3** | 0.20 ± 0.01 | 1.000 | 1.000 | 1.000 | -- | 0.002 |
| **P4** | 0.18 ± 0.01 | 0.008 | < 0.001 | < 0.001 | 0.002 | -- |
